# What helps, what hinders?—Focus group findings on barriers and facilitators for mobile service robot use in a psychosocial group therapy for people with dementia

**DOI:** 10.3389/frobt.2024.1258847

**Published:** 2024-06-21

**Authors:** Catharina Wasic, Robert Erzgräber, Manja Unger-Büttner, Carolin Donath, Hans-Joachim Böhme, Elmar Graessel

**Affiliations:** ^1^ Friedrich-Alexander-Universität Erlangen-Nürnberg (FAU), University Hospital Erlangen, Department of Psychiatry and Psychotherapy, Center for Health Services Research in Medicine, Erlangen, Germany; ^2^ Department of Artificial Intelligence/Cognitive Robotics, Faculty of Informatics/Mathematics, University of Applied Science Dresden (HTW Dresden), Dresden, Germany; ^3^ Independent Researcher, Dresden, Germany

**Keywords:** dementia, focus group, robot-assisted therapy, institutional care, user-centered design

## Abstract

**Introduction:**

Many countries are facing a shortage of healthcare workers. Furthermore, healthcare workers are experiencing many stressors, resulting in psychological issues, impaired health, and increased intentions to leave the workplace. In recent years, different technologies have been implemented to lighten workload on healthcare workers, such as electronic patient files. Robotic solutions are still rather uncommon. To help with acceptance and actual use of robots their functionalities should correspond to the users’ needs.

**Method:**

In the pilot study Care4All–Initial, we developed and field-tested applications for a mobile service robot in a psychosocial, multimodal group therapy for people with dementia. To guide the process and assess possible facilitators and barriers, we conducted a reoccurring focus group including people with dementia, therapists, professional caregivers as well as researchers from different disciplines with a user-centered design approach. The focus group suggested and reviewed applications and discussed ethical implications. We recorded the focus group discussions in writing and used content analysis.

**Results:**

The focus group discussed 15 different topics regarding ethical concerns that we used as a framework for the research project: Ethical facilitators were respect for the autonomy of the people with dementia and their proxies regarding participating and data sharing. Furthermore, the robot had to be useful for the therapists and attendees. Ethical barriers were the deception and possible harm of the people with dementia or therapists. The focus group suggested 32 different applications. We implemented 13 applications that centered on the robot interacting with the people with dementia and lightening the workload off the therapists. The implemented applications were facilitated through utilizing existing hard- and software and building on applications. Barriers to implementation were due to hardware, software, or applications not fitting the scope of the project.

**Discussion:**

To prevent barriers of robot employment in a group therapy for people with dementia, the robot’s applications have to be developed sufficiently for a flawless and safe use, the use of the robot should not cause irritation or agitation, but rather be meaningful and useful to its users. To facilitate the development sufficient time, money, expertise and planning is essential.

## 1 Introduction

Although demographic forecasts predict a constant or even declining prevalence of cognitive dysfunction in western societies (Wu et al., 2017; Nerius et al., 2020), the increasing global life expectancy will result in an overall larger number of older people with cognitive impairment (United Nations et al., 2020). Meanwhile, there is a workforce shortage in the healthcare sector, which is predicted to increase further (World Health Organization, 2006; OECD, 2016; World Health Organization, 2020).

The shortage of human resources as well as other factors such as working overtime, a high workload, demands for economic efficiency of health services, and increasing documentation puts stress on healthcare workers. The resulting outcomes are often burn-out, anxiety, depressive symptoms, a general decline in health, and the intention to leave the workplace–especially in long-term care facilities (Jacobs et al., 2016; Harrad and Sulla, 2018; Okuhara et al., 2021).

In recent years, certain processes, such as patient files and communications between healthcare actors, have been digitalized (Rahimi et al., 2018; Nadal et al., 2020); the use of robotic solutions in therapy on the other hand is still uncommon (Daum, 2017; Wasić et al., 2020; Seifert and Thilo, 2021).

Currently, most forms of dementia are not curable. The goal of interventions for care and therapy of people with dementia (PwDs) is therefore to provide the opportunity to participate in everyday life, maintain autonomy as well as the person’s different abilities, contribute to wellbeing and quality of life. Psychosocial therapies are recommended as one form of therapy for PwDs (Deuschl and Maier, 2016).

In the Care4All–Initial project, we used a mobile service robot to enhance group therapy for PwDs living in a long-term care facility. A reoccurring focus group discussed applications to be developed and reviewed the implementation to ensure that the use of the robot offered benefits for the attendees and therapists of the group therapy. Other published results of the project can be found elsewhere (Bahrmann et al., 2020; Dirks et al., 2022; Wasić et al., 2022).

The group therapy for PwDs followed the concept of MAKS therapy. It was conducted 3 to 6 days a week in the long-term care facility. The therapy group consisted of six to eight PwDs. MAKS is a multimodal psychosocial intervention consisting of four components, which are carried out in the same order in every session. The components include motor (M), everyday practical (A) and cognitive (K) exercises in a social setting (S). The MAKS therapy is manualized and evaluated, and each session lasts approximately 2 hrs. The activities of the therapy can be adjusted in their difficulty to adapt to the abilities of the therapy attendees appropriately. An in-depth explanation of MAKS therapy and its effects are published elsewhere (Graessel et al., 2011; Luttenberger et al., 2012a; Luttenberger et al., 2012b; Straubmeier et al., 2017; Gräßel, 2019; Luttenberger et al., 2019).

Since the robot we utilized in MAKS therapy did not have any software applications when the Care4All–Initial project started, we wanted to develop the robot’s functionalities by applying a user-centered design to identify needs and requirements and evaluate the design (cf. Norman, 2013). We chose a user-centered design approach as the robot was used in a specific setting with the potential goal of enhancing a therapy that was already proven effective (Straubmeier et al., 2017; Gräßel et al., 2019; Luttenberger et al., 2019). While the external appearance was set through the robot we bought, the functionalities were not. This gave us the opportunity to program the robot so it would meet the requirements of the attendees, the therapists and the therapy itself. The design process is an iterative one, starting with generating ideas, prototyping those ideas, testing them, observe the test and from those conclusions discuss the ideas again and develop them further. While this process can be used in different design approaches, user-centered design focuses on specific tasks, user demands, and aims to improve usefulness and usability of technical developments (Norman, 2013). A focus group is a group discussion using a moderator to guide the discussion about a set of defined questions or topics. The aim is not to find consensus, but to gather knowledge, experience and ideas from people with different fields of expertise (Krueger and Casey, 2000; Krueger, 2002). To implement the user-centered design approach we therefore decided to establish a reoccurring focus group for the duration of the project to serve as a starting point and to provide consecutive discussion partners for the robot’s development. The participants of the focus group were to represent every stakeholder impacted by using a robot in a group therapy for PwDs in a long-term care facility.

**FIGURE 1:**
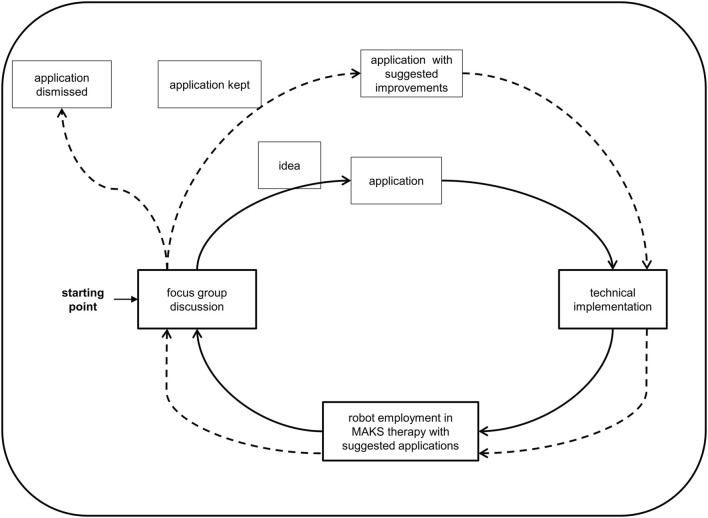
User-centered design for the project Care4All–Initial.

Figure 1 illustrates the process of the user-centered design.

Although technology is often used to compensate for abilities that are impaired due to symptoms of dementia (Hendriks et al., 2014; Lazar et al., 2017), recent research on human-machine-interaction turned towards focusing on using technology to support PwDs in meaningful ways: aiding communication and reminiscence via audio and video recordings (Hodge et al., 2019), engaging people with advanced dementia in activities and interactions through social robots (Raß et al., 2023), co-design gaming systems with PwDs (Suijkerbuijk et al., 2023), or evaluating the impact of videogame-based training for PwDs (Unbehaun et al., 2020). Furthermore, applying qualitative approaches can highlight the abilities of PwDs to communicate, co-create and recognize themselves and others (Kenning, 2018; Foley et al., 2019; Suijkerbuijk et al., 2019). These approaches can give justice to the dignity and personhood of PwDs.

For PwDs living in long-term care facilities, helpful technologies are multimedia computer programs, ambient assisted-living systems, or robots (Neal et al., 2020; Wasić et al., 2020). Robots are often used in therapy, to support activities of daily living, to help with physical tasks, monitor safety or medical conditions, or to keep them company (Broadbent et al., 2009b; Abdi et al., 2018; Wasić et al., 2020).

In projects using robots for PwDs, focus groups are usually conducted at the beginning of the project to establish goals for the project or to discuss design choices (Cooper et al., 2016; Korchut et al., 2017) or at the end to help interpret outcomes (Wu et al., 2014). While focus groups have the potential to involve PwDs in the design, research and evaluation of technology (Suijkerbuijk et al., 2019), they are not regular participants. Some studies choose to recruit medical doctors and professional caregivers (Korchut et al., 2017) or hospital and care facility managers (Radic and Vosen, 2020) instead. Other studies did involve older adults (Broadbent et al., 2009a) or people with mild cognitive impairment (Wu et al., 2012; Wu et al., 2014; Wu et al., 2016). Focus group studies have found that the robot needs to appear warm and friendly but must not look too human-like (Mori et al., 2012). It should be able to personalize its means of communication with the user (Wu et al., 2012; Cooper et al., 2016; Radic and Vosen, 2020). The focus groups desired functionalities that could support PwDs in their daily activities and could monitor falls, vital signs, and medical needs. For people living independently, the robot should help them maintain their independence or improve it (Broadbent et al., 2009a; Korchut et al., 2017). Privacy of PwDs’ data should be guaranteed, and isolation as well as a lack of human warmth should be prevented (Wu et al., 2016; Radic and Vosen, 2020). A focus group with people with mild cognitive impairment pointed out the stigma of using a robot as it may be perceived as a need for help and loss of independence (Wu et al., 2012; Wu et al., 2014; Wu et al., 2016). To our knowledge, there were no projects involving robots and PwDs that used focus groups for development, evaluation, and re-evaluation for the entire runtime of the project while involving different stakeholders–from PwDs, therapists, psychologists, medical doctors to care facility managers and informatics engineers.

This led to the following research question: Throughout the course of the project, what ethical concerns and application requests were asked for by stakeholders of the focus group? And which barriers hindered and what facilitated the usage of a robot in group therapy for PwDs?

Care4All–Initial was a cooperation between the department of artificial intelligence at the University of Applied Science Dresden (HTW Dresden), the neurological department of the University Hospital Dresden, the Center for Health Services Research in Medicine of the University Hospital Erlangen, the Cultus gGmbH, as well as Cognitec Systems.

## 2 Materials and methods

### 2.1 Design

The aim of the project Care4All–Initial was to create a robotic solution that would work and be used because it corresponds to the users’ needs. The aim of the reoccurring focus group was to provide insight into the users’ needs by giving ideas for robot applications, reviewing their implementation and discussing the ethical implications and concerns arising from using a robot for PwDs. The aim of this paper is to report on the discussion in the focus group meetings and how it influenced the design of the robot’s applications.

#### 2.1.1 Robot used and design approach for applications

**FIGURE 2:**
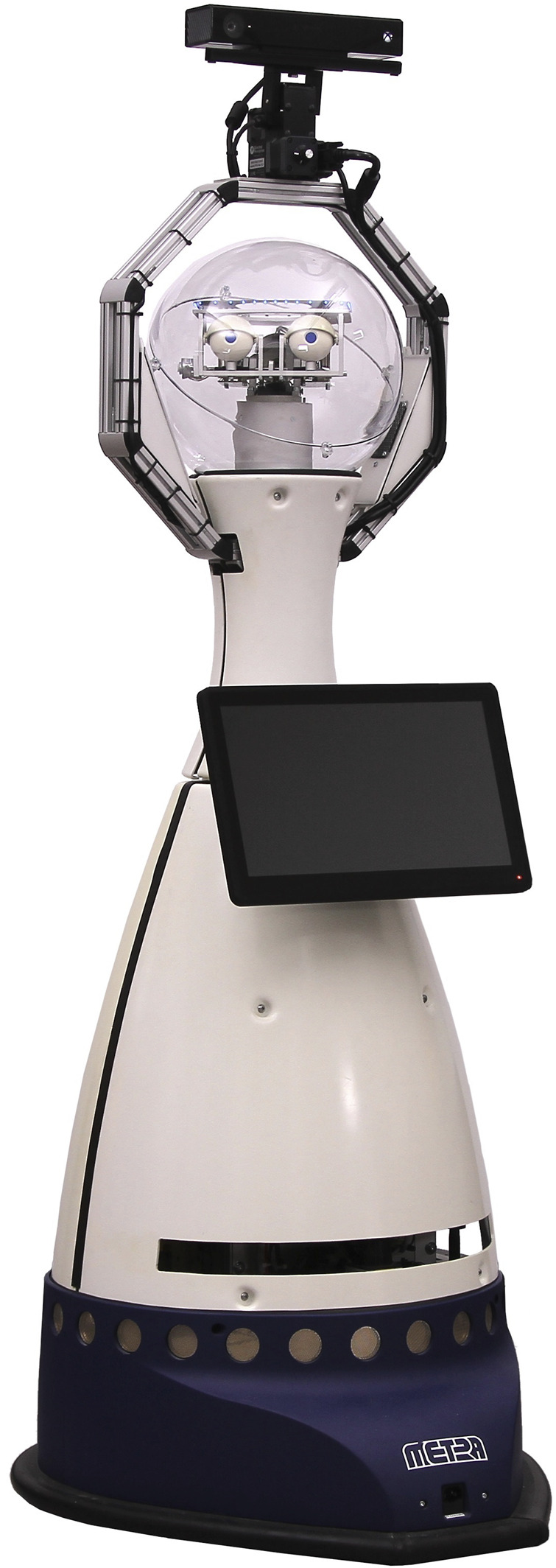
Robot Scitos G5 by MetraLabs.

We used a Scitos G5 by MetraLabs as the robotic platform with a Halma pawn-like body. The robot is roughly 1.80 m tall, weighs about 80 kg and has a 360° moveable head with two independently addressable eyes and eyelids to give a place to look at and facilitate interaction with the robot. It is equipped with a Kinect One, two laser range finders (forwards and backwards), a tactile sensor, and a touch pad (see Figure 2). Though it has eyes the robot is not designed to convey emotions using its hardware. We programmed the robot’s applications over the course of the project. At the start of the project, the robot had no software applications, which could be used within the MAKS therapy. The first applications were programmed after the first focus group meeting and then field-tested, before the second focus group meeting. Further applications as well as modification to existing ones were developed after the second focus group meeting and field-tested again. After the third focus group meeting two rounds of developments were performed and field-tested before the fourth focus group meeting. There were further modifications and developments after the fourth focus group meeting took place, but these are not addressed in this manuscript, as they were part of the follow-up study.

The focus group was the starting point for the iterative design approach (see Figure 1). Ideas for applications were gathered and discussed. The department of artificial intelligence at the HTW Dresden technically implemented these ideas. We tested the robot and its applications in the group therapy every few months and videotaped the robot in the field. The focus group evaluated the tested applications in the next meeting using video snippets. It was up to the focus group to decide what would happen to an application next: whether an application needed further development or not; whether it remained in the robot’s repertoire or was eliminated.

#### 2.1.2 Focus group design

Over the course of 2 years (September 2017 to June 2019), we held four focus group meetings with ten to twelve participants each. Due to the study period ending before the start of the COVID-19 pandemic, this did not interfere with the study. The first author conducted the focus group meetings as facilitator. At the time of the meetings, she held a Master’s degree in Gerontology and worked as a research assistant. She was trained to give presentations and to conduct focus group meetings via mock-up meetings.

Before each meeting, a moderator’s guide was written, containing several questions and corresponding prompts for the discussion (see Table 1). At the first focus group meeting the facilitator informed the participants about the proceedings and the privacy policy. She explained the focus group method, the basic rules for discussion, and the project’s aim. She explained her own role as facilitator and those of the note-takers as well as their affiliations. The facilitator opened and closed each meeting with brief questions that were answered by every participant (flash feedback method) and a brief summary of the last and current meeting, respectively.

**TABLE 1:** Overview of prompts used and questions asked in the focus group meetings.

	Date	Prompt	Question
focus group meeting	I: September 2017	Definition: robot, cognitive impairment	How are you associated with these terms? (FFM)
		Experiences with people with cognitive impairment (Think-Share)	**In which of these experiences could one employ a robot?**
		Discussion summary	**In which must one not employ a robot?**
			Personally, what is the best scenario to employ a robot? (FFM)
	II: January 2018	Presentation: MAKS therapy	What are your thoughts about MAKS therapy? Do you have any questions? (FFM)
		Explaining components of MAKS therapy	**How can the robot help with this component of the therapy? How can the robot support the therapists?**
		Discussion summary	Personally, which of the discussed application scenarios is the most interesting? (FFM)
	III: September 2018	State of the project at the 1-year mark	What are your experiences in this project so far? (FFM)
		Video snippets of tested application (Greeting, reading the news, telling jokes, Karaoke)	**How would you rate the tested application? Voting using cards (red = stop using application, yellow = application needs adaptation, green = application is good as is)**
		Discussion summary	**Why did you rate the tested application this way?**
			What priorities would you set for the further development of the robot? (FFM)
	IV:June 2019	Look back on project	In your opinion, what are the pros and cons of employing a robot in a long-term care facility? Did your opinion change over the course of the project? (FFM)
		Video snippets of tested application (Reading the news, short conversation while reading horoscope, controlling the robot via tablet, pictorial story, interactive game)	**How would you rate the tested application? Voting using cards (red = stop using application, yellow = application needs adaptation, green = application is good as is)**
		Experiences gained in the MAKS therapy, during the employment of the robot, and attending the focus group meetings	**Why did you rate the tested application this way?**
			Considering your experience, is the employment of a robot in the MAKS therapy feasible? Voting using cards (red = no, yellow = maybe, green = yes)
			Please explain your answer. (FFM)

Note. FFM, flash feedback method, every participant answers the question; Think- Share = first every participant takes notes on their own, and then ideas are collected.

The first focus group meeting centered on barriers and facilitators of the employment of a robot in a long-term care facility. The second meeting concentrated on using the robot in the MAKS therapy. The questions evaluating the robots applications in the third and fourth meeting always started with a video snippet of the respective application (with the faces of attendees and therapists blacked out and audio only of the robot’s voice) and a short voting by the participants holding up different colored cards to indicate, whether the application was good, bad or needed adaption. An open, detailed discussion of the application followed. The video snippet showed how the application, which was derived from the ideas of the focus group, was implemented in the field test and served as a memory for the focus group participants that were present in the MAKS therapy. The moderator made sure, to always ask for the opinion of the participant with dementia on each application and the suggested improvements.

Each moderator’s guide was pilot tested with external researchers to ensure the clarity of the questions. Questions and prompts were refined afterwards as necessary. We did not repeat the focus group meetings otherwise. After each meeting, the facilitator took field notes. Each meeting lasted about 2 h with a 10–15 min break. The focus group discussed the questions in the moderator’s guide. When a discussion about a topic ended, the facilitator summarized it and asked for further remarks and questions. Topics from past meetings were also brought up again in subsequent meetings as part of the iterative design approach. Both practices ensured data saturation and validity of the findings.

The study design and implementation was performed as specified by Krueger (2002) and Krueger and Casey (2000). We used content analysis (Mayring and Fenzl, 2019) as the underlying theoretical framework to code, condense, and report the data. This paper adheres to the COREQ reporting guidelines (Tong et al., 2007).

### 2.2 Participants

Before the project began, the cooperation partners discussed which stakeholders were directly or indirectly affected by the field-use of the robot and therefore needed to be represented in the focus group, like PwDs, therapists, professional caregivers, care facility managers, informatics engineers, etc. Table 2 gives an overview of the stakeholders we recruited and which focus group meetings they attended.

**TABLE 2:** Participants of the focus group meetings (September 2017 to June 2019).

Stakeholder	Present at focus group meeting (n)	Drop-out (reason) and re-recruitment
Certified nurse in the long-term care facility	I (1), II (1), III (1), IV (1)	-
MAKS therapist	I (1), II (1), III (2), IV (1)	-
MAKS attendee/long-term care facility resident	I (1), II (1), III (1), IV (1)	-
Relative of long-term care facility resident	-	yes (scheduling conflicts), no re-recruiting (scheduling conflicts)
Psychologist	I (1), II (1), III (1), IV (1)	yes (childbirth), re-recruiting at meeting III
Medical doctor	I (1), II (1), III (1), IV (1)	-
Informatics engineer	I (2), II (3), III (1), IV (2)	-
Quality manager in the long-term care facility	I (1), II (1), III (1), IV (2)	yes (prolonged illness), re-recruiting at meeting III
Residential sector manager in the long-term care facility	I (1), II (1), III (1), IV (1)	-
Ethicist	I (1), II (1), III (1), IV (1)	-
Data security engineer	I (1), II (1)	yes (termination of contract), no re-recruiting (position remained vacant)

Potential participants for the focus group were selected by purposive sampling to include the stakeholders from Table 2 and were contacted via email and telephone. We contacted 17 potential participants and were able to recruit at least one representative of each stakeholder group (14 in total). All participants were affiliated either to the long-term care facility or to other cooperation partners to some degree. Since the reoccurring focus group was part of a user-centered design we wanted to ensure that firstly, every stakeholder group was represented, hence the bigger sample size. Secondly, that the participants were involved in the ongoing development by deciding which of the scenarios were most important and will most likely have the biggest impact for the long-term facility through their various affiliations. Five participants had met the facilitator before the first focus group as they already cooperated with each other in the study. Before each participant’s first focus group meeting, they gave written consent to participate in the focus group and to publish the results in a summarized form. The Ethics council of the Friedrich-Alexander-Universität Erlangen-Nürnberg approved of the focus group and the project proceedings (252_18B).

Ten to twelve participants took part in each focus group meeting. Up to seven participants were female, and five were male. Up to six participants were affiliated with the long-term care facility; up to seven participants had a scientific background with up to three being informatics engineers. Up to six participants were directly affected by dementia (being a person with dementia or working with PwDs daily). The age range spanned from 25 to 77 years at the first focus group meeting. All participants had an urban background with all but one living in or near the city of the long-term care facility. Only one participant had been part of a focus group prior to the study and seven participants had experience in conducting research. Three participants had worked with a robot prior to the first focus group meeting and five participants had experience with the MAKS therapy. Four participants dropped-out for various reasons, of whom two could be re-recruited for the next focus group meeting (see Table 2).

### 2.3 Data collection

All focus group meetings were held on the grounds of the long-term care facility. Some participants were only willing to cooperate in the study and to participate in the focus group under the prerequisite that no audio or video files would be created. The research took place in a very sensitive surrounding–directly in their workspace or place of residents. Furthermore, under the prerequisite that taped statements might cause disadvantages in the future, answers in the focus group meetings would be less open, less authentic and less creative as shown in other studies (Flick, 1991; Gläser and Laudel, 2009; Rubin and Rubin, 2011). Therefore, an audio or video recording did not take place. Instead, the meetings were recorded in writing in real time by one to two nonparticipating researchers (an informatics engineer and a certified nurse) and the facilitator. No real names were used in the written recordings. Notes were taken with pseudonyms instead. Employing note takers from different fields ensured different perspectives in the written recordings. Because the facilitator and the note-takers were part of the research project, a bias must be assumed. Hence the usage of two independent written recordings from far-most stretch points of working fields to ensure a broader perspective within the resulting recording. Only researchers and participants were present during the meetings.

### 2.4 Data analysis

We transferred the written recordings into digital versions and used Microsoft Excel (2016) for a synoptic comparison. The data set was aggregated using the following steps: Firstly, each sentence was transferred into a digital version as written down by the note-takers. Secondly, the sentences were matched by using the pseudonyms of the participants. Resulting in sentences that were either recorded by one note-taker, or by more than one note-taker. The latter were re-worded by the first author taking the note-taker’s perspective into account. Because the note-takers were from different fields and used their respective vocabulary, the first author discussed the recordings with the note-takers during the process of aggregating them into one data set. This was done to prevent misinterpretation of the recordings. Lastly, some sentences that contained more than one topic or discussed different aspects of the same topic were split into half sentences. The first author then assigned categories to each sentence or half sentence (coding). Categories and subcategories were derived from the data (inductive approach), defined, and evaluated within the coding process consistent with content analysis (Mayring and Fenzl, 2019). Categories describe the topic of a sentence, which is specified furthermore in the subcategories. Sometimes the subcategories were assigned properties for further clarify the meaning, e.g., the category “reaction” was assigned to sentences that described the reaction of someone towards the robot or towards the group therapy and how the reaction was. The subcategories and their properties specified who reacted (e.g., a participant of the focus group or a resident of the long-term care facility) and how (e.g., neutral, negatively or positively). Sometimes sentences contained more than one category, but could not be split into further half sentences. For those combined categories were assigned, e.g., a statement on the robots adequate reaction to the residents was categorized as a requirement for a capability with the subcategory robot. The additional file Supplementary Table S1 shows the categories, subcategories and their properties as well as combined categories used for coding the data. The analysis of the focus group meeting was performed in the weeks directly after the meeting and completed before the next meeting was planned. The first author summarized the themes discussed by the focus group that emerged through the categorization of the data and wrote a report on each focus group meeting. As the coding and summarizing of the recordings was performed by only one researcher we sent the summary of each analysis to every participant for comments or corrections (cf. Harding and Whitehead, 2013). No participants made corrections. Because of the iterative nature of the study design and the lack of feedback to the summaries, the summaries of the previous focus group meetings were incorporated into the discussions of the following meetings. Firstly, by using them to start the focus group meeting to update every participant and have an equal starting point for the discussion. Secondly, themes from previous focus group meetings were used as prompts in the moderator’s guide to facilitate further discussion about them. We used the same analysis procedure of all four focus groups meetings and updated the categories, subcategories and combined categories when necessary.

We grouped the results into ethical concerns and applications. To summarize the ethical discussions, we sorted the concerns by the principles of biomedical ethics (Beauchamp and Childress, 2001). For the applications, we summarized the ideas and reviews and described the implementation process.

## 3 Results

The qualitative analysis resulted in three major themes: ethical concerns towards the use of a social robot in a long-term care facility, human-robot-communication as well as lightening the workload of professional caregivers.

### 3.1 Ethical concerns

Questions centering ethical concerns were explicitly prompted in the first two focus group meetings, although ethical concerns were discussed in every focus group meeting. Table 3 gives an overview of the discussed concerns sorted by the four principles of biomedical ethics (Beauchamp and Childress, 2001).

**TABLE 3:** Ethical concerns discussed in focus group sorted by the four principles of biomedical ethics (Beauchamp and Childress, 2001).

Principle	Discussed themes
Respect of autonomy	Use of data and biography; Respectful handling of scepticisms and refusal of robot; Voluntariness
Nonmaleficence	Liability; Transparent communication about robot, therapy and project; Appearance of robot and robotic voice; Deception of MAKS attendees due to interactions with the robot using Wizard-of-Oz control; Robot showing or handling emotions; Human-robot communication
Beneficence	Robot more than just entertainment; Robot useful for attendees and therapists; Public perception of employment of the robot; Support existing abilities of people with dementia
Justice	Participation in modern technology; Regularity of employment of the robot

#### 3.1.1 Respect for autonomy

The focus group discussed the use of MAKS attendees’ data and biographical information. Although a lot of the attendees’ information was already shared, because they were institutionalized, the robot employment was a new situation to which attendees and their legal proxies needed to give consent for their data to be used. The focus group agreed that respectful handling of skepticism towards the robot and project, the refusal to attend, the need for more information and voluntariness of participation at all times facilitate the employment of the robot.

#### 3.1.2 Nonmaleficence

Not harming the MAKS attendees or therapists in any way was a priority for the focus group. This encompassed liability, which needs clarification for the use of the robot outside of the project, and a transparent communication with therapists, attendees and their proxies. On that basis, the role and functionality of the robot and its employment, including it not being harmful to anyone involved, can be communicated to a more general public. As part of transparency, the appearance, voice, actions and behaviors of the robot should be such a way that it is without a doubt clear to any onlooker and listener that the robot is not a human being. It should be visible to the MAKS attendees that the robot is controlled by a human and does not act of its own agency. The focus group felt that the Wizard-of-Oz style of controlling the robot was deceptive and non-transparent but recognized its benefit within the scope of the project. As another aspect of the robot not appearing to be human, the focus group agreed on multiple occasions that showing a range of emotions and handling emotions of PwDs, especially negatively connoted emotions, must not be part of the robots functionalities and has to remain in the scope of the therapists and professional caregivers. The robot itself is not designed to show emotions by using its hardware, although it has eyes and furthermore uses a text to voice software. An application, where the robot showed emotion–the evaluation of news articles–was originally implemented to engage and activate the MAKS attendees in the activity and stimulate a conversation. After evaluating this application the focus group decided, that it should be discontinued and be replaced by an open-ended question. In this way, the robot could still potentially activate and engage the attendees, but would not show emotions.

#### 3.1.3 Beneficence

Although the focus group saw entertainment as a benefit for the therapy situation in itself, they agreed that the employment of the robot needed to add value and be meaningful for attendees and therapists in the MAKS therapy. To benefit the attendees, the robot should support their individual capabilities. The focus group noted that when supporting abilities, the individual capabilities as well as likes and dislikes of the PwDs needed to be considered. Integrating different degrees of difficulties of activities into the application to adapt to individual attendees could be a form of this individual support. Additionally, the needs and background of the MAKS attendees as a group need to be considered when using the robot. Some functionalities might be meaningful in one MAKS therapy group, while not being meaningful in another group, e.g., a former version of the MAKS therapy featured saying a Christian prayer together with the attendees, which is not suitable for a group of non-Christian attendees. To be useful to the therapists, the robot should decrease their workload and be perceived useful. The focus group stressed that the robot being beneficial to PwDs and therapists was also important for the public perception of the robot employment.

#### 3.1.4 Justice

Parallel to the principle of equality as basis for the principle of justice (Beauchamp and Childress, 2001) the focus group stated that participation in everyday life was a right of every individual. Employing the robot within the therapy gave the MAKS attendees an opportunity to participate in current technological advancements. To keep such participation meaningful, the robot had to be employed regularly. On the other hand, there was an injustice for the residents of the long-term care facility who did not attend the MAKS therapy sessions. They could not profit from the therapy itself or from the robot. Outside of the project, every resident should therefore be given the opportunity to participate in the therapy and to interact with the robot.

### 3.2 Applications

**FIGURE 3:**
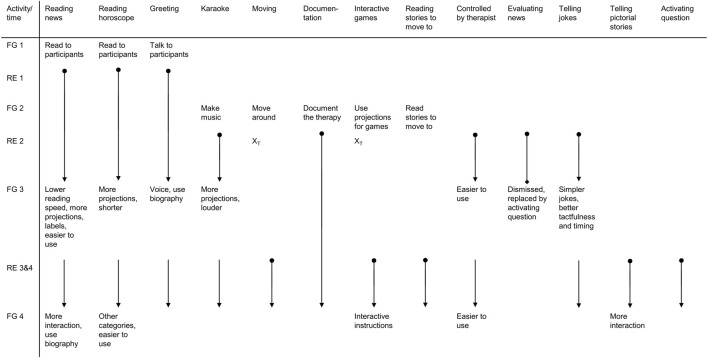
Implemented applications with reviews from focus group and development during the project Note FG = Focus group meeting, RE = robot employment, X_T_ = not implemented due to limited technical feasibility, 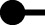
= start of the development, 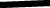
= continuation of development, 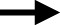
= discussed in focus group meeting, 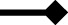
= development discontinued.

The focus group suggested 32 different applications for the robot. We implemented 13 (41%) of these during four robot employments. The applications can be sorted into human-robot-communication and functionalities lightening the workload of professional caregivers or therapists. Figure 3 provides a timeline of all implemented applications, their development and suggested improvements. It shows which ideas for applications were discussed in which focus group meeting, when the applications were first developed and field-tested in which robot employment as well as when they were discussed in the focus group meeting again including the suggested improvements. The additional Supplementary Table S2 gives an overview of the implemented applications, suggested improvements by the focus group, the overall acceptance by the focus group as well as the timeline when the application was discussed and when it was field-tested.

#### 3.2.1 Human-robot-communication

The focus group discussed how the robot could communicate with the MAKS attendees. Utilizing different channels of perception (e.g., voice, projection) could benefit attendees with hearing impairment or cognitive dysfunction. The focus group stressed that it was important for the robot’s voice to be clear and articulate. In eight applications, we used IVONA software to convert text to speech for the robot’s voice and soft- and hardware for projection. The volume and speed of the voice could be changed on the spot. The speech synthesis had problems with the pronunciation of foreign and borrowed words as well as names. The focus group criticized the monotonous tone and missing emotions in the robot’s voice, and the occasional unsuitable stressing of the words. Since the attendees of the MAKS therapy have a cognitive impairment, the robot should communicate directly by using their names and reacting to certain situations (e.g., greeting the participant and introducing itself). Preferably, the robot would move closer to the attendee who it is talking to in order to capture and hold their attention.

At the start of the therapy, the robot greeted the attendees and introduced itself. In later employments of the robot, it compared the faces of the attendees with photos in a database and greeted them by their names. During the therapy, the robot would read news, their horoscope, a story to move to (e.g., raise a hand when a certain word is mentioned) and a pictorial story (Max and Moritz) to the attendees, play karaoke, and tell jokes. However, it proved difficult to find jokes that were appropriate for the audience. The focus group also criticized the timing and tactfulness of the jokes.

The news were sourced from a local newspaper. All MAKS attendees were locals to the region of the long-term care facility. The therapists would see a list of news articles on their tablet and could select, which one the robot should read aloud. This way it was possible to feature articles about interests of individual attendees, like their favorite sports team. The focus group suggested to label the news articles so that the MAKS therapists would know, if an article was of interest for one of the attendees or if it had been read already.

For the MAKS therapy group in the project Care4All- Initial a part of the social component of the therapy was changed: Instead of saying a Christian prayer together with the attendees, the group read their horoscope. All of the MAKS group attendees were non-Christians or non-practicing Christians. But reading the horoscope was a pastime already established in the long-term care facility, which is why we integrated it into the therapy for the duration of the project. One participant of the focus group initially voiced concern about reading a horoscope to PwDs, since it is an activity that focuses on the future. The MAKS attendee and therapist, who were part of the focus group as well, explained that it was rather for the enjoyment and provided a prompt for communication and friendly humor within the therapy sessions. Within the project Care4All the manual for the MAKS therapy was revised and does now contain different activities for the social component that can be adapted to fit an individual MAKS therapy group (Gräßel, 2019).

We tried having the robot move around the therapy room, but this was problematic due to the small size of the room. The attendees would have to stand up and move their chairs out of the way for the robot to be able to move around. In the follow-up project it was possible to use a different, bigger therapy room for the MAKS therapy and have the robot move around.

To stimulate conversation, we had the robot evaluate news articles after reading them aloud (e.g., “That sounds sad”). However, the MAKS attendees did not react to the robot’s statements, and the robot’s evaluations often failed to match the news appropriately. Furthermore, in this instant the robot was showing emotions, which was something the focus group had ruled out in prior meetings. The focus group therefore decided to replace it by an activating question. After reading a news article, the robot would now ask, “What do you think of that?” The MAKS therapist would then go on to moderate and stimulate a discussion about the news article.

From the second robot employment onwards, a video projector was used in the therapy and its usage was successively expanded to enhance the applications with projections: we added corresponding pictures and captions to news, horoscopes, the karaoke application as well as the pictorial story Max and Moritz.

**FIGURE 4:**
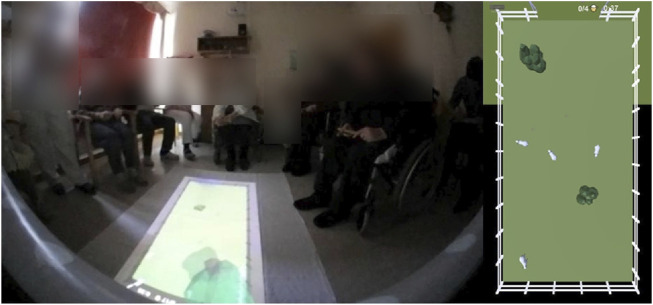
Interactive game with sheep, live implementation (left) and overview (right).

Lastly, we developed an interactive game, where a pasture with sheep was projected onto the floor. The MAKS attendees had to make loud noises to drive the sheep through a fence (see Figure 4). The robot was equipped with a directional microphone to detect where the noise was coming from. The attendees had problems being loud enough on their own, but instruments helped resolve this issue. Due to a lack of funding for the researcher who developed the interactive game, we could only field-test the interactive game once.

#### 3.2.2 Lighten workload of professional caregivers

The focus group discussed how to lighten the workload on the MAKS therapists by automatically documenting the therapy and giving the therapists the possibility to rest their voice. For the robot to be controlled by the therapists, the instructions and control interface needed to be clear and easy to understand.

In the first employment, members of the department of artificial intelligence controlled the robot from outside the therapy room. From the attendees’ perspective, the robot appeared to act autonomously (Wizard-of-Oz). From the second employment onwards, the MAKS therapists themselves controlled the robot via a tablet in the therapy room. The therapists experienced problems using the tablet. They had to pay a great deal of attention to controlling and were not able to focus enough on the attendees. The focus group emphasized in later meetings that controlling the robot needed to be effortless for the therapists and that the control therefore needed to be simplified and partially automated.

We also tested automated documentation of the therapy by the robot for the use in the attendees’ electrical health record. The robot could document attendance and log used applications. Compatibility issues and data privacy requirements occurred with the interface between the robot and the electronic health record and documentation was therefore suspended. Another idea to support the MAKS therapists was to use speech to control the robot or dictate text for the electronic health record, which we did not implement during the pilot study. In the follow-up study we were able to solve the interface problem and have a documentation of the therapy that is compatible to the attendees’ electrical health record.

The applications focusing on the robot interacting with the attendees could also be helpful to the therapists. While the robot read to or sang with the attendees the therapists could concentrate on the attendees rather than on the task, or take a moment of rest within the 2 hour long therapy. The therapists would also use these applications to keep the group engaged, while they would help one of the attendees, e.g., with using the restroom. Some improvements to the applications, like labeling the news articles according to the interests of attendees, could also help with lightening the mental load of the therapists.

#### 3.2.3 Non-implemented applications

We did not implement 19 of the suggested 32 applications, mostly due to ethical concerns or a lack of technical feasibility. The additional Supplementary Table S3 provides an overview of the non-implemented applications. In the follow-up project, we were able to implement five additional applications from the original suggestions: compatibility with the electronic health record, the MAKS attendees communicating with the robot using signs or button boxes, the robot providing cognitive exercises, the robot instructing the attendees on motor exercises and the robot as a sitting vigil (Erzgräber et al., 2022).

We could not implement several applications because necessary hardware or software (e.g., enhancing the room with fragrance) was not available, would not fit into the robot or might cause a problematic build-up of heat. For other suggested applications, we did not have the necessary use rights (e.g., cognitive and motor exercises for PwDs). Furthermore, we could not implement applications where the robot would learn on its own through interactions with the MAKS attendees because of the timeframe of the project. Initiation of the interactive learning process coincidences with mistakes of the robot, which disrupt therapy and possibly irritate or agitate the PwDs. Three of the suggest applications raised ethical concerns by the focus group (aiding with food intake, handling aggressions, and measuring emotions) and four other applications did not fit into the MAKS therapy (monitoring falls/sitting vigil, dancing, suggesting games outside of the therapy, and waking attendees up) and were therefore not implemented.

## 4 Discussion

We conducted a focus group with four consecutive meetings to develop applications for a mobile service robot in a group therapy for PwDs and to answer the research questions: Throughout the course of the project, what ethical concerns and application requests were asked for by stakeholders of the focus group? And which barriers hindered and what facilitated the usage of a robot in group therapy for PwDs?

Regarding ethical concerns, the points stressed by the focus group in all meetings were data privacy and the voluntariness of participation (respect for autonomy), transparency, acting without deceiving PwDs or their proxies (nonmaleficence), the robot’s usefulness to MAKS attendees and therapists (beneficence), and the opportunity for the PwDs to participate in technical advancements (justice).

Regarding application requests, the focus group suggested 32 different applications, of which 13 were implemented, adjusted and developed and one was eliminated after the field trial. Facilitators and barriers for the usage of the robot are discussed in the following.

### 4.1 Ethical facilitators and barriers

Ethical aspects that facilitate the employment of a robot centered on respect for autonomy, participation of PwDs and the usefulness of the robot. The focus groups requests aligned with published ethical guidelines, like the 1964 Helsinki Declaration (WMA, 2008). The focus group stressed that autonomy and participation of PwDs in technology should be handled the same beyond the research project. Moreover, preparing and updating informational documents, consent forms and data security concepts in coordination with different authorities and the ethics council and designing them as accessible as possible for all stakeholders facilitates the use of the robot. Although the time and expertise needed for preparation and updating these documents can act as a barrier, when it is underestimated, and delays the project work.

As a facilitator the focus group stressed the need for the robot to be useful for the MAKS attendees and the MAKS therapists. This is consistent with findings on factors influencing the acceptance of technology and robot acceptance in healthcare services: The more the intended users perceived the robot as useful, the more they accepted it (Heerink et al., 2009; Heerink et al., 2010; Holden and Karsh, 2010; Rahimi et al., 2018). Furthermore, the aspect of a helpful robot for PwDs did not mean to compensate lost abilities through technology. The focus group rather emphasized that the robot and its functionalities should provide opportunity for PwDs to use their existing abilities. Long-term research on the MAKS therapy for people with cognitive impairments shows, that consistent therapy can help to keep cognitive abilities and abilities of everyday life on a constant level (Graessel et al., 2011; Straubmeier et al., 2017). A robot providing interactions tailored to the individual capabilities of a person with dementia may have a similar effect if used regularly, while being meaningful. Studies on assistive technology found, that meaningful interactions of PwDs with the technology can facilitate the successful incorporation of it (Arntzen et al., 2016).

The usefulness of the robot for the therapists lies in its ability to lighten their workload, e.g., through taking on some of the documentation of the therapy or providing moments of rest for the therapists within in the 2 hour long MAKS therapy. A study using an effort-scale to assess the effort the MAKS therapists felt directly before and directly after the therapy session during a robot employment indicated, that the average effort was lower after the session compared to before. All 5 MAKS therapists worked in the same long-term care facility and were not part of any research team. Over the course of 4 weeks of robot employment the average effort declined even further (Dirks et al., 2022).

In addition, the change in the activity of the social component of the MAKS therapy (horoscope instead of Christian prayer) helped to improve the therapy manual to include more options to tailor the social aspect of the MAKS therapy to the group, their background and their likings.

The focus group saw ethical barriers to the implementation of a robot in the vulnerability of the PwDs and MAKS therapists. Neither should be harmed by the robot employment in any way whether trough deception (with the Wizard-of-Oz control), nor through fear of being taped or possibly being replaced by a robot. Other authors also viewed the Wizard-of-Oz control as non-transparent (Unger-Büttner, 2019). However, if the robot is not allowed to learn through trial and error, since this could cause irritation or agitation in PwDs, the Wizard-of-Oz style could be used instead (Dow et al., 2005; Weiss et al., 2009). Surveys found similar fears for healthcare workers who had a negative attitude toward robots and perceived the employment of the robot as hazardous (Turja et al., 2018; Rebitschek and Wagner, 2020).

Contrary to other findings, the focus group did not indicate that the robot could be stigmatizing. None of the participants of the focus group reported of MAKS attendees and therapists or residents, their relatives and employees of the care facilities stating that the robot was stigmatizing to PwDs. On one hand, other authors reported that a robot was seen as a sign of dependence, and therefore perceived as stigmatizing by people with cognitive impairment (Wu et al., 2016; Demange et al., 2018). On the other hand, within the Care4All–Initial project, the robot was used in group therapy for PwDs who were residents of a long-term care facility. The stigma of being dependent that affects PwDs (Lion et al., 2019) might already be at play because of their place of residence (Nolan et al., 2006; Dobbs et al., 2008). Though we did not find an indication for an existing stigmatization, it can still be present and needs to be studied more in-depth in future employments of the robot. Similarly, an overly enthusiastic view of the robot and its applications could raise expectations the robot cannot meet leading to frustration. Moreover, it could influence application development to a point that alienates less enthusiastic possible users.

In one instance, the focus group contradicted itself. On the one hand, the focus group stressed that the appearance and voice of the robot should make clear that the robot is not a human being. This request was in line with their demand for transparency and their feeling that the PwDs should never be deceived by it. Even further, the focus group agreed, that the robot was not to show or handle emotions. They had the news evaluating application replaced, because the robot showed emotions when in use. On the other hand, the application for telling jokes was kept in the robot’s repertoire. Furthermore, the focus group criticized the voice of the robot for being too monotonous, for not conveying emotions, lacking timing and tactfulness, and for sometimes stressing words inappropriately. This criticism centered on the PwDs sometimes not being able to understand the robot properly, because of the synthesis of the robot’s speech. Other authors have also reported problems with the synthesis of a robot’s speech, which has led to decreases in the feasibility of the robot’s employment (Gerling et al., 2016; Kouroupetroglou et al., 2017; Salatino et al., 2017). This contradiction could be an “as-if” problem (Unger-Büttner, 2019). Even though the focus group knows that the robot’s voice is synthesized the expectations for the voice’s quality in communication were as high as if it had been a human voice.

It can be further argued, that the request of the focus group for the robot to clearly appear non-human and not convey emotions is contradicted by the robot itself by having eyes and using its voice for communication–especially when it comes to humor while telling jokes or a range of emotions when evaluating news articles. Studies show that humor in human-robot-interaction can lead to a more favorable perception of the robot overall (Oliveira et al., 2021), especially for robots with human-like features (Zhang et al., 2021). Concerning jokes told by robots, other authors report differences on how well the jokes were perceived according to themes (Tay et al., 2016) and age of participants (Bechade et al., 2016). These findings correspond to the focus group’s want to keep the joke application for its potential benefit to enable humor and human-robot-interaction but to adapt it to better fit the therapy attendees.

The findings on ethical concerns discussed by the focus group participants helped us to refine our ethical framework and actions for the development and employment of the robot in the field. On multiple occasions, we provided information and informational material about the project and its proceedings to the PwDs, their proxies as well as the MAKS therapists. With the enactment of the General Data Protection Regulation (GDPR) by the European Union (European Union, 2016) in 2018, we updated the informational documents and consent forms. The findings also formed a basis to revise the manual and materials for the MAKS therapy. We developed and adapted the robot’s functionalities after consulting the focus group or the MAKS therapists and did so according to their ideas. A detailed depiction of all implemented applications including their development and improvements over the course of the project can be found in Figure 3. The topics of how to best communicate the robot’s employment to the public, who is liable for any damages to the robot or caused by the robot, and the possibility of stigmatization through the robot employment remain to be researched more in-depth.

### 4.2 Facilitators and barriers to application implementation

Sufficient time, funding and employees facilitate the development and implementation of applications for the robot. We could build on existing applications for the robot movement and control and used the same software for speech synthesis and hardware for projections for several applications to save time and money. The importance of funding is highlighted by the interactive game, which could only be field-tested once, as the programming researcher could not be employed further due to limited funds.

The use of the video projector gave us the chance to further develop applications by incorporating images and text as the robot did not have any built-in hardware to do so. The focus group stressed that using multiple channels of communication will facilitate communication between the robot and the MAKS group attendees. Without the video projector the robot could have only used text to speech software to communicate with MAKS attendees, which would be a barrier to MAKS attendees with hearing impairments or cognitive impairments regarding processing spoken words. Furthermore, we were able to develop an interactive game that relied on a projection.

Barriers to the implementation of the applications were missing hardware, incompatible software, missing use rights, and an insufficient or problematic way of adapting the applications. Applications that function reliably and would not irritate or agitate the PwDs were crucial and other studies reported that a malfunctioning robot resulted in decreased feasibility and increased agitation in the PwDs (Gerling et al., 2016; Hebesberger et al., 2016; Moyle et al., 2016; Servaty et al., 2020; Koh et al., 2021). The focus group criticized the voice of the robot, because the PwDs were not able to understand the robot properly sometimes. Other authors have reported similar problems, which has led to decreases in the feasibility of the robot’s employment (Gerling et al., 2016; Kouroupetroglou et al., 2017; Salatino et al., 2017).

Although the social robot was not able to use any of the applications designed to assist in the MAKS therapy autonomously, its presence could still have an impact on MAKS attendees as well as on MAKS therapists. Different studies showed that social robots in long-term care facilities can facilitate communication and engagement of PwDs (Koutentakis et al., 2020) with acceptance of the robot increasing, when it was acting human-like in its communication (Whelan et al., 2018). The impact of the social robot and its applications on MAKS therapists and MAKS attendees are published elsewhere (Bahrmann et al., 2020; Dirks et al., 2022).

### 4.3 Strengths and limitations

In the Care4All–Initial project, we conducted a reoccurring focus group consisting of all stakeholders affected by a robot-assisted therapy for PwDs that guided the entire developmental process of actually useful robot applications. An additional strength of the project was that we were able to implement 41% (and additional 15% in the follow-up project) of all suggested applications and employed the robot in 25 therapy sessions, which is high considering the short project duration of 2 years. The implemented applications ranged in their complexity from reading the news to an interactive game. Furthermore, only applications suggested by the focus group members were developed making them the center of the design process. The implemented applications were reviewed continuously on their usefulness and ethical implications by a diverse group of stakeholders including PwDs, professional caregivers, informatics engineers, MAKS therapists and others.

The employment of the robot and the focus group were conducted in only one long-term care facility, the note-takers were part of the research team and the content analysis was performed by only one researcher. A bias must therefore be assumed. Nevertheless, the findings correspond in large parts with the results from other studies and present a holistic view of the employment of a robot in group therapy for PwDs. Due to the nature of qualitative research, the effects of the robot-assisted therapy on MAKS attendees and MAKS therapists compared to a standard MAKS therapy remain to be assessed in following studies. Recruiting the focus group members from a different long term care facility or a different region could have resulted in different suggestions for applications, although the main themes of ethical concerns, human-robot-communication and lightening the workload of professional caregivers would likely have been the same.

## 5 Conclusion

Using a reoccurring focus group to provide guidance in a user-centered design to develop robot applications for a psychosocial group therapy for PwDs consisting of all stakeholders affected, we identified barriers and facilitators. From our findings, we draw the following suggestions for our future research and for designing robots used in group therapy for PwDs in general:

• First, the robot and its applications should be developed sufficiently enough to ensure that no harm is done to the users and it can be used flawlessly. PwDs might react differently than expected even in familiar situations. Therefore, engineers need to develop situations with extreme operating parameters (edge cases) to ensure a secure operating around PwDs. This includes the general safety of PwDs and therapists. In the field, the use of the robot should not initiate any irritating or agitating situations being active or inactive.
• Second, the robot needs to be useful and beneficial to its users to successfully employ it. To identify beneficial and meaningful use cases a user-centered design with the users and the different involved professions should be considered. An eye-level exchange between all involved stakeholders furthermore encourages understanding and respect for different needs, concerns, capabilities and personal autonomy. This benefits the acceptance of the developmental process and of the robot overall.
• Third, to technically develop applications for robots time, money, components, and qualified employees should be considered. To save time and money, new applications should be built on existing ones or should use similar hardware or software. The necessary hardware should fit the robot, and software with compatible interfaces should be used. Use rights, data security needs and concepts should be considered and dealt with beforehand.

## Data Availability

The datasets for this article are not publicly available due to concerns regarding participant anonymity. Requests to access the datasets should be directed to the corresponding author.

## Supplementary material

Supplementary Table S1
Categories, subcategories and their properties as well as combined categories used for coding the data.

Supplementary Table S2
Overview of the implemented applications, suggested improvements by the focus group, the overall acceptance by the focus group as well as the timeline when the application was discussed and when it was field-tested.

Supplementary Table S3
Overview of the non-implemented applications.

## Abbreviations

MAKS, Group therapy for people with dementia consisting of motoric, cognitive and everyday activities in a psychosocial setting; PwDs, People with dementia.
