# Supplementary material for: What helps, what hinders?—Focus group findings on barriers and facilitators for mobile service robot use in a psychosocial group therapy for people with dementia
Source: Front Robot AI. 2024 Jun 21;11:1258847. doi: 10.3389/frobt.2024.1258847 (PMC11224299; doi:10.3389/frobt.2024.1258847)
Supplement: Supplementary file 1 [file Table1.pdf]

Supplementary Material

What helps, what hinders? – Focus group findings on barriers and facilitators for mobile service robot use in a psychosocial group therapy for people with dementia

Wasic, Catharina*, Erzgräber, Robert, Unger-Büttner, Manja, Donath, Carolin, Böhme, Hans-Joachim, Graessel, Elmar

*** Correspondence:**
Catharina Wasic: catharina.wasic@uk-erlangen.de

Supplementary Table S1

Categories, subcategories, combined categories, and definitions used to code the protocols

Category	Definition	Subcategory	Combined categories
Requirements	The requirements within the project; Subcategories to define what or who is required	robot/<br>dementia/<br>project/<br>confidence/<br>therapist/<br>controlling the robot/<br>transparency	Requirement for profit/<br>Requirement for doubt/<br>Requirement for capability/<br>Requirement for reaction/<br>Requirement for customization
Profit	Profit to the project; Subcategories for what or who profits	through therapy/<br>through the robot/<br>science	Profit through requirements/<br>Profit through features
Consideration	Consideration two opposite poles or opinions	none	Discussion (from Consideration and Doubt)
Capability	Capabilities that each group possesses	none	
Rejection	Topics/ matters that need to be rejected	none	
Doubt	Uncertainty about something;	profit/	Doubts about capabilities

	Subcategories for what is in doubt	handling/ implementation/ protection of data	
Characteristic	Characteristics of something or someone	none	Characteristic that creates doubt
Taboo subject	Subjects that are not part of the content of the project	none	
Possibility	State or fact of being possible or likely; Subcategories for what is possible	deployment of the robot/ therapy/ application (name of application)	Possibility of support
Reaction	Described reaction to the robot or therapy; Subcategories to describe who is reacting in what way	Who: resident/ employee/ relative/ focus group participant/ public/ science how: positive/ negative/ critical	Handling of the project (from reactions and keeping the balance)
Customization	Altering an existing fact or application to better fit the situation Subcategories to describe what is being customized or for whom	What: therapy/ robot For whom: attendees	
Complications	Descriptions of problems that occurred; Subcategories to detail with what complications arise	therapy/ robot/ applications/ controlling the robot	Complications from characteristics
Improvement	Improvements to the robot and its applications;	voice/ pictures/ presentation/ karaoke/ jokes/	

	Subcategories to describe the suggested improvements	newspaper/<br>horoscope/<br>evaluating news/<br>controlling the robot/<br>administration
Change of mind	Change of mind in the process of the project by a participant;<br>Subcategories to describe direction of mind change	none/<br>pro-robot/<br>anti-robot
Planning	Planning for future matters either in the project or after the project;<br>Subcategories for follow-up projects and subject of planning	Follow-up project<br><br>subject: operation/<br>voice
Request/ Answer	Either request for clarification or short answer to request without additional information	none
Comparison	Comparison of one thing with another;<br>Subcategories to describe the content of the comparison	MAKS therapy versus MAKS therapy with robot
