# Supplementary material for: What helps, what hinders?—Focus group findings on barriers and facilitators for mobile service robot use in a psychosocial group therapy for people with dementia
Source: Front Robot AI. 2024 Jun 21;11:1258847. doi: 10.3389/frobt.2024.1258847 (PMC11224299; doi:10.3389/frobt.2024.1258847)
Supplement: Supplementary file 2 [file Table2.pdf]

*Supplementary Material*

# What helps, what hinders? – Focus group findings on barriers and facilitators for mobile service robot use in a psychosocial group therapy for people with dementia

**Wasic, Catharina*, Erzgräber, Robert, Unger-Büttner, Manja, Donath, Carolin, Böhme, Hans-Joachim, Graessel, Elmar**

*** Correspondence:**
Catharina Wasic: catharina.wasic@uk-erlangen.de

**Supplementary Table S2**

*Implemented applications*

| Application | Acceptance by FG | Suggested Improvement | Further ideas | Discussed in FG | Employed in RE |
|---|---|---|---|---|---|
| Reading News | +/~ | improve voice; adjustable reading speed; using pictures and labels for categories | use of biography and interactive element to choose article | I, II, III, IV | I, II, III, IV |
| Reading Horoscope | +/~ | suitable source of horoscope; interactive conversation about zodiac sign | use other categories; easier to use | I, II, III, IV | I, II, III, IV |
| Greeting | ~ | improve voice; use of portrait for face recognition | use of biography for individual conversation | I, II, III, IV | I, II, III, IV |

| | | | | | |
|---|---|---|---|---|---|
| Karaoke | +/~ | adjustable playback speed and voices; sorted by genre | none | II, III | II, III, IV |
| Robot moving | ~ | better handling of space restrictions | none | II | III |
| Documentation | ~ | implement dictating and picture taking function and weekly review | compatibility with electronic patient record; measuring reaction time of attendees | II | II |
| Interactive games | +/~ | adjust sensitivity of microphone and sensors; adjustable difficulty | implement game tutorial; self-adjusting levels of difficulty | II, IV | IV |
| Stories to move to | + | none | none | II | III |
| Therapist controls robot | ~ | make interface easier to use | automate applications | III, IV | II, III, IV |
| Evaluating news | - | dismissed | none | III | II |
| Jokes | ~ | use jokes appropriate for people with cognitive impairment | none | III | II, III, IV |
| Pictorial story | + | implement interactive element using rhymes | none | IV | IV |
| Activating question | + | address individual attendees | initiate short conversations | IV | IV |

*Note*. FG = Focus group; RE = employment of the robot; + = accepted; ~ = mixed acceptance; - = not accepted
