# Supplementary material for: What helps, what hinders?—Focus group findings on barriers and facilitators for mobile service robot use in a psychosocial group therapy for people with dementia
Source: Front Robot AI. 2024 Jun 21;11:1258847. doi: 10.3389/frobt.2024.1258847 (PMC11224299; doi:10.3389/frobt.2024.1258847)
Supplement: Supplementary file 3 [file Table3.pdf]

Supplementary Material

What helps, what hinders? – Focus group findings on barriers and facilitators for mobile service robot use in a psychosocial group therapy for people with dementia

Wasic, Catharina*, Erzgräber, Robert, Unger-Büttner, Manja, Donath, Carolin, Böhme, Hans-Joachim, Graessel, Elmar

*** Correspondence:**
Catharina Wasic: catharina.wasic@uk-erlangen.de

Supplementary Table S3

Non-implemented applications and reasons

Reason	Application	Implemented in follow-up study
technical issues	attendees communicating with the robot;	yes
	compatibility with electronic patient file;	yes
	going for a walk outside;	no
	robot learning through interaction;	no
	robot realizing helplessness and differentiating it from laziness;	no
	virtual walk through the city;	no
	enhancing the room with fragrance;	no
	motor exercises;	yes
	cognitive exercises;	yes
	individual documentation;	no
	virtual household chores;	no
	using speech to control robot;	no
ethical concerns	aiding food intake;	no

	handling aggression;	no
	measuring emotions	no
not part of MAKS therapy or project	monitoring falls/ sitting vigil;	yes
	suggesting games to attendees outside of the therapy;	no
	waking attendees up;	no
	dancing	no
